# Risk Factors for Chronic Cough in Young Children: A Cohort Study

**DOI:** 10.3389/fped.2020.00444

**Published:** 2020-08-12

**Authors:** Yin To Au-Yeung, Anne B. Chang, Keith Grimwood, Yolanda Lovie-Toon, Michelle Kaus, Sheree Rablin, Dan Arnold, Jack Roberts, Sarah Parfitt, Jennie Anderson, Maree Toombs, Kerry-Ann F. O'Grady

**Affiliations:** ^1^Australian Center for Health Services Innovation@ Centre for Healthcare Transformation, Queensland University of Technology, South Brisbane, QLD, Australia; ^2^Child Health Division, Menzies School of Health Research, Tiwi, NT, Australia; ^3^Department of Respiratory Medicine, Queensland Children's Hospital, South Brisbane, QLD, Australia; ^4^School of Medicine, Griffith University, Southport, QLD, Australia; ^5^Menzies Health Institute Queensland, Griffith University, Southport, QLD, Australia; ^6^Department of Paediatrics, Gold Coast Health, Southport, QLD, Australia; ^7^Caboolture Community Medical, Caboolture, QLD, Australia; ^8^Carbal Health Services, Toowoomba, QLD, Australia; ^9^UQ Rural Clinical School, The University of Queensland, Toowoomba, QLD, Australia

**Keywords:** acute respiratory illness, chronic cough, children, childcare, cohort study

## Abstract

**Background and Objective:** Data on the predictors of chronic cough development in young children are scarce. Our primary objective was to examine the factors associated with young children developing a chronic cough, with a focus on childcare attendance.

**Methods:** A secondary analysis of data collected in a prospective cohort study of children presenting to three emergency departments and three primary healthcare centers in southeast Queensland, Australia. Eligible children where those aged <6-years presenting with cough and without known underlying chronic lung disease other than asthma. Children were followed for 4 weeks to ascertain cough duration. The primary outcome was persistent cough at day-28. Logistic regression models were undertaken to identify independent predictors of chronic cough including sensitivity analyses that accounted for children with unknown cough status at day-28.

**Results:** In 362 children, 95 (26.2%) were classified as having chronic cough. In models that included only children for whom cough status was known at day-28, symptom duration at enrolment, age <12 months [adjusted odds ratio (aOR) 4.5, 95% confidence interval (CI) 1.1, 18.7], gestational age (aOR 3.2, 95%CI 1.4, 7.9), underlying medical conditions (aOR 2.6, 95% CI 1.3, 5.5), a history of wheeze (aOR 2.6, 95% CI 1.4, 4.8) and childcare attendance (aOR 2.3, 95% CI 1.2, 4.4) were independent predictors of chronic cough. Amongst childcare attendees only, 64 (29.8%) had chronic cough at day-28. The strongest predictor of chronic cough amongst childcare attendees was continued attendance at childcare during their illness (aOR = 12.9, 95% CI 3.9, 43.3).

**Conclusion:** Gestational age, underlying medical conditions, prior wheeze and childcare attendance are risk factors for chronic cough in young children. Parents/careers need to be aware of the risks associated with their child continuing to attend childcare whilst unwell and childcare centers should reinforce prevention measures in their facilities.

## Introduction

Cough, one of the most common reasons for primary healthcare attendances in the community (1), is a key symptom of acute respiratory illnesses (ARIs). ARIs impose a substantial health burden on children, their families and the healthcare sector (1–3). When cough persists for ≥4-weeks, it is defined as chronic cough (4). While chronic cough may reflect uneventful delayed resolution, it may also signal an underlying chronic respiratory disorder (4, 5). A recent study reported that amongst Australian children who presented to an emergency department (ED) with ARI with cough, one in five developed chronic cough and, of those reviewed by a respiratory physician, 31% were diagnosed with a previously undetected chronic respiratory disorder (e.g., asthma, tracheobronchomalacia, aspiration disorder, or asthma) (6).

Thus, chronic cough post ARI is important and knowing, at the point of the acute presentation, who are at risk of developing chronic cough will be clinically useful to clinicians and parents. Intervening early in the transitional stage from acute to chronic cough improves cough outcomes (7), and may reduce the burden on families (8) and the healthcare sector (2). However, although primary-care studies have reported 10% of children with an ARI are still coughing after day-25 (9), there are little data on the predictors of chronic cough in children. In our previous study, restricted to a single low-socioeconomic site, predictors of chronic cough were age <12-months, eczema, childcare attendance, previous history of chronic cough, parental Indigenous status, and low income (10). However, generalizability of these data is limited.

Risk factors for childhood ARIs include childcare attendance, now common in the modern era (10–17). In 2017, >40% of Australian children aged <5-years attended childcare on a daily basis (18). However, specific risk factors for ARI with cough amongst childcare attendees remain poorly defined, including whether childcare attendees are at higher risk of developing chronic cough. A Finnish cohort study of 894 children identified those attending daycare facilities had higher mean days per month of ARI symptoms compared to children attending home care and family daycare (5.5, 4.9, and 4.8 days, respectively, *p* = 0.03) (16). A United States cross-sectional study involving 3,000 children (17) reported the odds of ARI symptoms correlated with time spent in daycare; children who attended ≥20-h/weeks had higher odds for a range of ARI symptoms than those who attended <20-h/weeks [adjusted odds ratios (aOR) ranged from 1.50 to 1.88] (17)

There is a research gap in predictors of chronic cough in children presenting with an ARI. Also, amongst Australian children there are no data addressing the role of childcare in chronic cough developing post-ARI. Thus, our primary objective was to determine what factors are associated with a cough lasting at least 4-weeks in preschool children with ARI.

## Materials and Methods

### Design

This was a secondary analysis of data collected in a multi-center cohort study of children aged <15-years presenting to healthcare with cough. The full study protocol was published previously (19). Here, we included children aged <6-years and data from the first 4-weeks of follow-up. Data after this time-point were not included as children with chronic cough at that time point were randomized to an intervention, the results of which have been published (7).

### Setting

Recruitment occurred between July 2015 and October 2018 in South-East Queensland, Australia, in three primary healthcare services and three hospital EDs. The primary healthcare services included one outer urban and two regional communities and their client population was predominantly Aboriginal and Torres Strait Islander people. The EDs included two regional hospitals (in the same areas as two of the primary healthcare services) and the Queensland Children's Hospital (Brisbane), Queensland's only tertiary pediatric hospital. Only children presenting to EDs with triage categories 4 or 5 (lowest acuity) were included to reflect children who could otherwise have presented to primary healthcare.

### Study Participants

In this analysis, we included children aged <6-years (i.e., pre-schoolers) presenting with cough as a symptom who had no known underlying chronic lung disease other than asthma. Children with asthma were included given inconsistencies with the diagnosis of asthma in young children (20). Exclusion criteria included: immunosuppressive conditions; receiving immunomodulating drugs for >2-weeks in the preceding 30-days; current or planned participation in another intervention study during the 8-weeks of follow-up in the overall study; severe ARI requiring admission, and; insufficient English that inhibited informed consent or collection of study data. Parents of children were approached by trained research officers at the time of presentation. Written informed consent was obtained following provision and a plain language statement that explained the study in detail.

### Data Collection

Demographic, epidemiological, and clinical data were collected at baseline via interviewer administered questionnaire and medical record review. Weekly follow-up was conducted via email and/or phone interview with parents/carers. Data collected at follow-up included cough duration, type and severity, and childcare attendance. Three attempts were made each week to contact parents/carers; two consecutive weeks of failed contacts resulted in the child being classified as lost-to-follow-up (LTFU).

We classified a child as being a childcare attendee on at enrolment on the basis that parents reported a minimum of 1-day/week attendance at a childcare facility. Type of childcare (i.e., home care, family daycare or childcare center) and frequency of attendance on a weekly basis were collected.

We defined chronic cough as no break in cough over the 4-weeks follow-up period. A break in cough was defined as absence of cough for at least three consecutive days and nights as per previous studies (2, 21). Chronic cough was classified as “no” if at any time point the parent reported the cough had stopped for ≥3 consecutive days and nights and classified as “yes” if there was no break in cough reported at all four of the weekly follow-up time-points. Amongst children with data missing for at least one timepoint, chronic cough was classified as unknown if there were no other timepoints where the parent/career reported the cough had stopped. Chronic cough was classified as “no” if it was known the cough had stopped prior to LTFU, otherwise it was classified as unknown. Childcare attendance whilst cough persisted was defined as at least 1-week where the parent/career reported at follow-up that the child had attended childcare the previous week and there had been no break in cough.

### Sample Size

As this was a secondary analysis of data collected in a larger study, a sample size calculation was not undertaken.

### Statistical Analysis

Univariate analyses compared baseline characteristics between (a) children who did and did not develop chronic cough (b), children who did and did not attend childcare, and (c) those attending childcare who did and did not develop chronic cough. Comparisons of proportions were performed using the χ^2^ test or χ^2^ test for trend; comparisons of means were undertaken using *T*-tests, and the Kruskall-Wallis test was used to compare medians. Variables with a *p* < 0.1 in univariate analyses were selected for inclusion in logistic regression models; age, gender and the Indigenous status of the child and were retained in all models. The primary models excluded children with unknown cough status at day-28. Sensitivity analyses were then undertaken in which children with unknown cough status at day-28 were classified as “cough stopped” and then “cough not stopped.” Variables with a *p* < 0.05 were considered independently associated with the outcome of interest. Adjusted odds ratios (aORs) were reported with the corresponding 95% confidence intervals (95%CI). All analyses were undertaken in Stata 15 (StataCorp LLC, Texas, USA).

### Ethics Approval

The Human Research Ethics Committees of the Queensland Children's Hospital (HREC/15/QRCH/15) and the Queensland University of Technology (1500000132) approved the study.

## Results

### Study Cohort and Demographics

**Figure 1:**
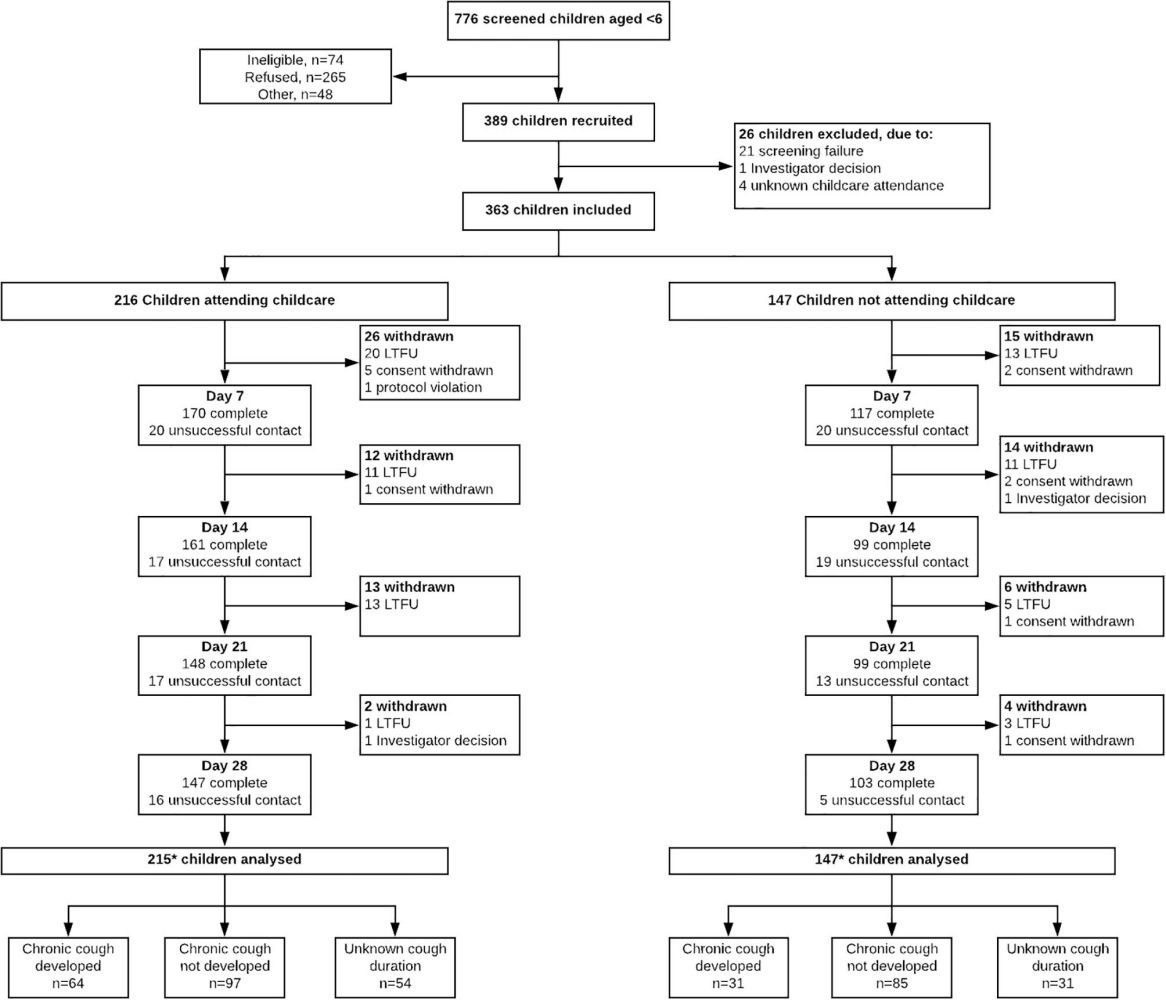
Flow diagram of screening, enrolment and follow-up of children aged <6-years presenting with cough as a symptom to three primary health care services and three hospital emergency departments.

Between July 2015 and October 2018, 776 children aged <6-years were screened and 389 children enrolled (Figure 1). There were no differences between children enrolled and not enrolled by season of screening, sex and age. Of the 389 enrolled children, 362 were included in the analysis (Figure 1). The median age was 19 months (interquartile range (IQR): 11–36); 56.2% male, and 59.5% attended any childcare service on a regular basis. Cough status at day-28 was unknown in 85 children; the baseline characteristics of children who did and did not have a known cough status at day-28 are presented in Supplementary Table 1. Compared to children for whom cough status was known at day-28, children with unknown cough status were more likely to have a previous diagnosis of any respiratory illnesses (aOR = 1.90, 95% CI 1.1, 3.2) and a mothers with a post-school education (aOR = 1.90, 95% CI 1.1,3.2), and less likely to have mothers of an older age (aOR = 0.95, 95% CI 0.90, 0.99) and private health insurance (aOR = 0.47, 95% CI 0.90, 0.99). The baseline characteristics of children who did and did not attend childcare are in Supplementary Table 2. Factors independently associated with childcare attendance are in Supplementary Table 3.

### Predictors of Chronic Cough Development at Day-28

Of those for whom cough status was known at day-28 (*n* = 277), 95 (34.3%) children were classified as having chronic cough. Chronic cough prevalence at day-28 in all children was 26.2% (95/362). Differences in selected characteristics between children by cough status are presented in Table 1.

**Table 1:** Selected baseline characteristics comparing children with and without CC, *n* = 277 (%).

**Characteristic**	**All children (*n* = 277)**	**Chronic cough** **Yes** **(*n* = 95)**	**Chronic cough** **No** **(*n* = 182)**	***p*-value**
**ENROLMENT SITE**				
Brisbane	154 (55.6)	46 (48.4)	108 (59.3)	0.216
Caboolture	89 (32.1)	36 (37.9)	53 (29.1)	
Toowoomba/Warwick	34 (12.3)	13 (13.7)	21 (11.5)	
**AGE AT ENROLMENT (MONTHS)**				
<12	75 (27.1)	29 (30.5)	46 (25.3)	0.219
12– <24	96 (34.7)	34 (35.8)	62 (34.1)	
24– <60	85 (30.7)	29 (30.5)	56 (30.8)	
≥60	21 (7.5)	3 (3.2)	18 (9.9)	
Median (IQR) months	19 (11–36)	18 (10–31)	20 (11–37)	
**GENDER**				
Female	122 (44.0)	46 (48.4)	76 (41.8)	0.289
Male	155 (56.0)	49 (51.6)	106 (58.2)	
**CHILD'S INDIGENOUS STATUS**				
Non-Indigenous	198 (71.5)	65 (68.4)	133 (73.1)	0.415
Indigenous	79 (28.5)	30 (31.6)	49 (26.9)	
**SEASON OF ENROLMENT**				
Spring	70 (25.3)	25 (26.3)	45 (24.7)	0.037
Summer	32 (11.6)	6 (6.3)	26 (14.3)	
Autumn	70 (25.3)	19 (20.0)	51 (28.0)	
Winter	105 (37.9)	45 (47.4)	60 (33.0)	
**GESTATIONAL AGE AT BIRTH**				
37+ weeks	250 (90.3)	79 (83.2)	171 (94.0)	0.004
<37 weeks	27 (9.7)	16 (16.8)	11 (6.0)	
**BIRTHWEIGHT**				
2,500+ grams	258 (93.1)	86 (90.5)	172 (94.5)	0.214
<2,500 grams	19 (6.9)	9 (9.5)	10 (5.5)	
**COUGH DURATION (DAYS)**				
<14	204 (73.7)	60 (63.2)	144 (79.1)	<0.001
14– <28	29 (10.5)	9 (9.4)	20 (11.0)	
≥28	42 (15.2)	26 (27.4)	16 (8.8)	
Declined/unknown/ missing^**^	2 (0.7)	0 (0.0)	2 (1.1)	
**HISTORY OF CHRONIC COUGH**				
No	185 (66.8)	50 (52.6)	135 (74.2)	<0.001
Yes	86 (31.1)	44 46.3)	42 (23.1)	
Declined/unknown/ missing	6 (2.1)	1 (1.1)	5 (2.7)	
**DIAGNOSED WITH ANY RESPIRATORY ILLNESS IN THE PAST 12 MONTHS**				
No	136 (49.1)	42 (44.2)	94 (51.7)	0.223
Yes	140 (50.5)	53 (55.8)	87 (47.8)	
Unknown^**^	1 (0.4)	0 (0.0)	1 (0.5)	
**PARENT REPORT OF ASTHMA DIAGNOSIS IN THE PAST 12 MONTHS**				
No	252 (91.0)	86 (90.5)	166 (91.2)	0.851
Yes	25 (9.0)	9 (9.5)	16 (8.8)	
**ITCHY RASH DURATION** **>6-MONTHS**				
No	245 (88.5)	79 (83.2)	166 (91.2)	0.022
Yes	30 (10.8)	16 (16.8)	14 (7.7)	
Unknown/missing	2 (0.7)	0 (0.0)	2 (1.1)	
**ECZEMA HISTORY**				
No	230 (83.0)	73 (76.8)	157 (86.3)	0.030
Yes	41 (15.5)	21 (22.1)	22 (12.1)	
Declined/unknown/ missing	4 (1.4)	1 (1.1)	3 (1.6)	
**HISTORY OF WHEEZING/WHISTLING**				
No	119 (42.9)	26 (27.4)	93 (51.1)	<0.001
Yes	2155 (56.0)	69 (72.6)	86 (47.3)	
Declined/unknown/ missing^**^	3 (1.1)	0 (0.0)	3 (1.7)	
**HISTORY OF OTHER MEDICAL CONDITIONS**				
No	225 (81.2)	67 (70.5)	158 (86.8)	0.002
Yes	51 (18.4)	27 (28.4)	24 (13.2)	
Unknown/missing^**^	1 (0.4)	1 (1.1)	0 (0.0)	
**NUMBER OF OTHER CHILDREN LIVING IN THE HOUSE**				
0	79 (28.5)	35 (36.8)	44 (24.2)	0.068
1–2	164 (59.2)	48 (50.5)	116 63.7)	
3+	34 (12.3)	12 (12.6)	22 (12.1)	
**ATTENDS CHILDCARE**				
No	116 (41.9)	31 (32.6)	85 (46.7)	0.024
Yes	161 (58.1)	64 (67.4)	97 (53.3)	
**PRIVATE INSURANCE COVERING CHILDREN**				
No	164 (59.2)	60 (63.2)	104 (57.1)	0.430
Yes	108 (39.0)	34 (35.8)	74 (40.7)	
Declined/unknown/ missing	5 (1.8)	1 (1.0)	4 (2.2)	
**EXPOSURE TO TOBACCO SMOKE**				
No	196 (70.8)	65 (68.4)	131 (72.0)	0.537
Yes	81 (29.2)	30 (31.6)	51 (28.0)	
**DIAGNOSIS AT TIME OF ED OR CLINIC PRESENTATION**				
Asthma	19 (6.9)	6 (6.3)	13 (7.1)	0.593
Bronchiolitis	18 (6.5)	6 (6.3)	12 (6.6)	
Croup	14 (5.1)	5 (5.3)	9 (5.0)	
Lower respiratory tract infection	9 (3.3)	1 (1.1)	4 (4.4)	
Pneumonia	6 (2.2)	0 (0.0)	6 (3.3)	
Upper respiratory tract infection	146 (52.7)	53 (55.8)	93 (51.1)	
Other	31 (11.2)	11 (11.6)	20 (11.0)	
Not documented	34 (12.3)	13 (13.7)	21 (11.5)	

** *Category has been excluded from p-value calculation*.

In the primary analysis that included only children in whom cough status at day-28 was known, in addition to symptom duration at enrolment, age <12 months (aOR 4.5, 95% CI 1.1,18.7), gestational age at birth (aOR = 3.2, 95% CI 1.3,7.9), underlying medical conditions (aOR = 2.6, 95% CI 1.3, 5.5), a history of wheeze (aOR = 2.6, 95%CI 1.4, 4.8 and childcare attendance (aOR = 2.3 (95% CI 1.2, 4.4) were independent predictors of chronic cough (Table 2).

**Table 2:** Predictors of the presence of chronic cough at day-28 following presentation for an ARI with cough in children aged <6-years (excluding children for whom cough status at day-28 was unknown), *n* = 274.

	**Adjusted odds ratio**	95% CI^*^	***p*-value**
Age at enrolment			
60+ months	Reference		
24– <60 months	1.8	0.4–7.7	0.414
12– <24 months	2.9	0.7–12.2	0.143
0– <12 months	4.5	1.1–18.7	0.041
Male gender	0.6	0.4–1.1	0.133
Indigenous child	1.3	0.7–2.5	0.428
Duration of first symptom (days)			
<7	Reference		
7 - <14	2.6	1.2–5.6	0.015
14+	2.7	1.4–5.1	0.002
Wheeze in past 12 months	2.6	1.4–4.8	0.002
Other medical conditions^**^	2.6	1.3–5.5	0.010
Gestational age <37 weeks	3.2	1.3–7.9	0.011
Attends childcare	2.3	1.2–4.4	0.016

* *CI, confidence interval*.

** *medical conditions were parent-reported: recurrent tonsillitis (23), chronic ear disease (14) congenital birth defects (13), heart disease (9), neurological diseases (5), diabetes (1), kidney disease (1)*.

In the sensitivity analysis in which unknown cough status was classified as cough stopped, all of the above remained independently associated with chronic cough at day-28 however childcare was no longer statistically significant (aOR 1.65, 95% CI 0.92, 2.95). When unknown cough status at day-28 was classified a chronic cough, factors no longer statistically associated with chronic cough were age <12 months (aOR 2.0, 95% CI 0.79, 4.96), presence of other medical conditions (aOR 1.7, 95% CI 0.92, 3.07) and gestational age <37 weeks (aOR 2.1, 95% CI 0.96, 4.56).

### Predictors of Chronic Cough Development at Day-28 in Childcare Attendees Only

Amongst the 215 childcare attendees, 64 (29.8%) had chronic cough at day-28, 97 (45.1%) had stopped coughing, and 54 (25.1%) had unknown cough status. Univariate analyses of attendee characteristics associated with chronic cough are presented in Supplementary Table 4. The results of regression models, including sensitivity analyses and adjusting for age, sex and Indigenous status, for children attending childcare only are presented in Table 3. Amongst only those children for whom cough status at day-28 was known duration of symptoms at enrolment, a prior history of chronic cough, indicators of allergy (intermittent itchy rash and pets at home), maternal influenza vaccination during pregnancy and continued attendance at childcare whilst unwell with a cough were independent predictors of chronic cough at day-28.

**Table 3:** Predictors of chronic cough at day-28 in childcare attendees, excluding and including children with unknown cough status.

	**Adjusted odds ratio**	95% CI^*^	***p*-value**
**Children with known cough status (*****n*** **=** **159)**			
Duration of symptoms at enrolment			
<7 days	Ref		
7– <14 days	4.2	1.2–15.2	0.030
14+ days	5.1	1.9–13.2	0.001
Prior history of chronic cough	2.9	1.2–7.5	0.023
History of intermittent itchy rash in past 12 months	4.7	1.0–22.0	0.05
Maternal influenza vaccine in pregnancy	4.5	1.7–11.6	0.002
Pets at home	4.0	1.6–9.8	0.003
Attended childcare with cough during follow-up period	12.9	3.9–43.3	<0.001
**Children with unknown cough status classified as cough stopped prior to day-28 (*****n*** **=** **213)**			
Duration of symptoms at enrolment			
<7 days	Ref		
7– <14 days	1.7	0.5–5.6	0.363
14+ days	3.6	1.5–8.4	0.003
Maternal influenza vaccine in pregnancy	3.9	1.6–9.7	0.003
Pets at home	3.2	1.4–7.3	0.006
Attended childcare with cough during follow-up period	26.1	8.0–84.5	<0.001
**Children with unknown cough status classified as chronic cough (*****n*** **=** **213)**			
Duration of symptoms at enrolment			
<7 days	Ref		
7– <14 days	3.0	1.1–8.1	0.025
14+ days	3.1	1.5–6.5	0.003
History of intermittent itchy rash in past 12 months	4.2	1.2–14.5	0.025
Maternal influenza vaccine in pregnancy	2.3	1.1–4.6	0.023
Prior history of chronic cough	2.4	1.3–4.7	0.009
Pets at home	2.2	1.2–4.2	0.017

* *CI, Confidence interval*.

Of the 64 childcare attendees who developed chronic cough, only five (7.8%) were absent from childcare over all 4-weeks of their illness; nine (14.1%) attended every week (Table 4).

**Table 4:** Weekly attendance at childcare by cough persistence amongst childcare attendees for whom contact was successful each week and complete data on both cough and childcare attendance were available.

	**Cough persisted 1-week**			**Cough persisted 2-weeks**			**Cough persisted 3-weeks**			**Cough persisted 4-weeks**		
	**Complete data** **=** **165/215**			**Complete data** **=** **152/215**			**Complete data** **=** **133/215**			**Complete data** **=** **128/215**		
	**Yes**	**No**	**Total**	**Yes**	**No**	**Total**	**Yes**	**No**	**Total**	**Yes**	**No**	**Total**
Absent from Childcare	89 (60.1)	5 (29.4)	94 (57.0)	15 (17.2)	6 (9.2)	21 (13.8)	10 (16.3)	7 (9.9)	17 (12.8)	18 (32.7)	7 (9.6)	25 (19.5)
Attended Childcare	59 (39.9)	12 (70.6)	71 (43.0)	72 (82.8)	59 (90.8)	131 (86.2)	52 (83.9)	64 (90.1)	116 (87.2)	37 (67.3)	66 (90.4)	103 (80.5)
Total children per week	148 (89.7)	17 (10.3)		87 (57.2)	65 (42.8)		62 (46.6)	71 (53.4)		55 (44.0)	73 (58.4)	

## Discussion

In 362 Australian children aged <6-years enrolled across six centers, we found 26% developed chronic cough. Independent predictors for developing chronic cough post-ARI with cough were younger age, childcare, longer cough duration at enrolment, a history of wheeze in the past 12 months and of other medical conditions and being born preterm. Amongst childcare attendees, predictors of chronic cough were having: a prior history of chronic cough, a household pet; a longer duration of illness at enrolment; prior intermittent itchy rash; antenatal influenza vaccination and continued attendance at childcare whilst ill. These factors did not change substantially when children for whom cough status at day-28 was unknown were reclassified as either chronic cough or cough resolved in sensitivity analyses. The proportion of children with persistent cough each week who continued to attend childcare was high.

When including all children in the analysis, the prevalence of chronic cough post-acute cough in our study is similar to the 20–26% identified in other studies in South-East Queensland using the same methods and similar communities (6, 10). Our study supports the findings of those studies with respect to risk factors for chronic cough (10, 22), including preterm birth, illness duration at presentation and childcare. Preterm birth has been associated with a range of both acute and chronic respiratory illnesses (23, 24). The role of comorbidities in contributing to the development of chronic cough post-acute-illness is not well-studied. The relationship between childcare and increased risk of ARI has been reported previously (11, 15, 16, 25). Childcare has also been associated with respiratory morbidity in children with chronic neonatal lung disease (26) and protracted bacterial bronchitis (27). However, there are limited data evaluating the role of childcare in chronic cough development.

Childcare in countries such as Australia is now necessary for many families. Thus, we examined the predictors amongst the subset of childcare attendees; the strongest association (aRR = 12.9, 95% CI 3.9, 43.9) was continued attendance at childcare despite cough persistence. Only five (7.8%) children who developed chronic cough had been absent their entire 4-weeks illness. This may reflect parental decision making on attendance given their need to return to work and/or challenges with communicable disease control guidelines in childcare facilities. An Australian qualitative study examined parental disease prevention beliefs for sending children to childcare when unwell (28). Choices were made in the context of family/societal obligations, peer expectations and needing to work, which was influenced by perceived illness severity (28). Indeed, gastrointestinal illnesses were considered more serious than colds and influenza despite parents recognizing infections can be spread by coughing (28). We also found a higher proportion of employed mothers among childcare attendees. Previous studies report that because of work schedules, employed parents send unwell children to childcare (15, 28). Complete withdrawal of a child with persistent cough from childcare until the cough has resolved is unlikely to be feasible or acceptable for many families and therefore heightened attention to infection control practices in childcare facilities in the instance of a child with persistent cough is necessary to protect both the unwell child as well as other children in the facility.

Among childcare attendees, having household pets and signs of atopy were associated with chronic cough. Several studies support the association between atopy and ARI (10, 29), but the evidence for the relationship between pet exposure and asthma and allergic diseases in childhood is conflicting (30, 31). The association between chronic cough and antenatal influenza vaccination is difficult to explain; we could not identify any biologically plausible mechanism. A possible explanation is that working mothers may be more likely to receive the influenza vaccine, including antenatally, and to send their child to childcare. Thus, the finding must be interpreted with a high level of caution, particularly given the established benefits of influenza vaccine during pregnancy to infant health (32), including a reduction in laboratory confirmed influenza early in infancy (32) as we did not verify vaccination status with medical records and recall bias is likely.

It is beyond the scope of our study to address the likely complex mechanistic actions of the risk factors we have identified in this study in the development of chronic cough. These mechanisms will likely vary depending on the child and his/her socio-economic status and environment. The respiratory tract and nervous systems of children undergo a series of maturation processes that produce the cough neural circuits (33). Pathological processes, particularly infection and inflammation, during this period lead to altered cough responses that may become persistent (33).

Approximately 28% of children had a cough of duration of more than 2-weeks at time of presentation to the GP or ED and 38% of these children had persistent cough at day-28. The most common diagnoses at time of presentation for these children were upper respiratory tract infections (64%), conditions other than those specifically described in Table 1 (11%) and in 12% a diagnosis was not documented in the medical record. Our previous studies indicate children with cough may have multiple presentations to healthcare providers for their cough before presenting to an emergency department (8, 34). This reflects parental concern that the cough is not resolving and also the impact on parent and child health-related quality of life, particularly given financial concerns, cough severity and whether cough is occurring during the day, at night or both (8). That the majority had a diagnosis of upper respiratory tract infection highlights the need for parents and healthcare providers to be aware that a third of children presenting with more than 2-weeks of cough may develop chronic cough and seek appropriate review at the 4-weeks timepoint.

The strengths of our study are the multi-center approach, the comprehensive data collected and the follow-up of children for 4-weeks post-presentation to healthcare with cough to capture cough status. However, our study has limitations, particularly the proportion of children with missing weekly contact data and unknown cough status at day-28 and that we did not adjust the analyses for multiple comparisons. The missing data reflects the challenges of capturing busy parents with young children. However, the sensitivity analyses did not substantially change our findings. It is plausible that loss to follow-up would be more likely in children whose cough has stopped, particularly given the known burden of cough on families (8, 35)and that parents in the study were informed at enrolment that if their child had persistent cough at day-28 they would be reviewed by a pediatrician over the following weeks (19).

We used 4-weeks of cough to define chronic cough as per almost all pediatric chronic cough guidelines [summarized in Table 2 of a systematic review (36)] including the American (37) and Australian guidelines (38). Four weeks of cough incurs significant burden on the child and family and early intervention improves outcomes. Children in the primary study on which this paper is based (7) with chronic cough at day-28 were subsequently randomized to an intervention and followed for a further 4-weeks, the outcomes of which have been published previously (7). Amongst children randomized to the control group (*n* = 58), 51% of children with known cough outcome (*n* = 45) continued to cough for 4-weeks, while cough duration was unknown in 13/58 (22%) due to loss to follow-up. Loss to follow-up was however equal between the treatment and control arms.

Further, we did not collect at baseline the duration that children had been attending childcare prior to enrolment and thus were unable to evaluate length of time in childcare before enrolment with cough outcomes. Nevertheless, the proportions of chronic cough and unknown cough status identified are similar to our other studies in the same settings. Additionally, as we did not perform serial microbiological testing, those with sequential respiratory infections may have been misclassified as chronic cough. Finally, parent/career recall bias is also a potential limitation, particularly given our finding with respect to antenatal influenza vaccination.

Of all the factors significantly associated with chronic cough, childcare attendance is arguably the most modifiable. It is a known risk factor for ARI and here we have shown that it adversely impacts ARI outcomes in children. Our study highlights the risks associated with continued childcare attendance whilst unwell, particularly for those children with increased vulnerability to disease. However, given childcare is a necessity for some, parents need to be informed of these risks and helped to identify potential strategies to ameliorate risk if ongoing attendance is unavoidable. This may include parents working with their childcare provider on potential physical distancing strategies in the center and facilitating close attention to the child and career's cough and hand hygiene. From a public health perspective, further work is needed to address the structures and operations of childcare centers to minimize risk, including commitment to infection control guidelines.

## Data Availability Statement

The datasets generated for this study are available on request to the corresponding author.

## Ethics Statement

The studies involving human participants were reviewed and approved by The Human Research Ethics Committees of the Queensland Children's Hospital (HREC/15/QRCH/15) and the Queensland University of Technology (1500000132) approved the study. Written informed consent to participate in this study was provided by the participants' legal guardian/next of kin.

## Author Contributions

YA-Y developed the research question, undertook all data analysis and wrote the first, and final draft of the manuscript. AC and KG provided substantial input into the design and implementation of the primary study and the manuscript. YL-T, MK, SR, DA, and JR played a major role in study implementation, data quality assurance, and assistance to YA-Y with data analysis. JA and MT contributed to the design of the primary study and oversaw its implementation in primary care. K-AO'G conceived the original study and the research question for this paper, supervised and trained YA-Y and assisted with the preparation of the manuscript. All authors contributed to and approved the final manuscript.

## Conflict of Interest

K-AO'G, AC, and KG received a National Health & Medical Research Council Project Grant related to this project. AC was an author of the UptoDate chapters on chronic cough in children. The remaining authors declare that the research was conducted in the absence of any commercial or financial relationships that could be construed as a potential conflict of interest.

## Abbreviations

ARI: acute respiratory illness
aOR: adjusted odds ratio
aRR: adjusted relative risk
CI: confidence interval
ED: emergency department
IQR: inter-quartile range
LTFU: lost to follow-up.
