# Supplementary material for: Risk Factors for Chronic Cough in Young Children: A Cohort Study
Source: Front Pediatr. 2020 Aug 12;8:444. doi: 10.3389/fped.2020.00444 (PMC7435047; doi:10.3389/fped.2020.00444)
Supplement: Supplementary file 1 [file Table_1.DOCX]

***Supplementary Table 1 – Baseline characteristics table of the study population, comparing children who did and did not attend childcare, n = 363***

| Characteristic | All children (n=363) | Children attending childcare (n=216) | Children not attending childcare (n=147) | P-value |
| --- | --- | --- | --- | --- |
| Enrolment site |  |  |  |  |
| Brisbane | 205 (56.5) | 126 (61.5) | 79 (38.5) | **0.001** |
| Caboolture | 116 (32.0) | 76 (65.5) | 40 (34.5) |  |
| Toowoomba / Warwick | 42 (11.6) | 41 (33.3) | 28 (66.7) |  |
| Season on enrolment |  |  |  |  |
| Spring | 89 (24.5) | 58 (65.2) | 31 (34.8) | 0.649 |
| Summer | 43 (11.9) | 24 (55.8) | 19 (44.2) |  |
| Autumn | 89 (24.5) | 52 (58.4) | 37 (41.6) |  |
| Winter | 142 (38.1) | 82 (57.8) | 60 (42.3) |  |
| Age at enrolment |  |  |  |  |
| <12 months | 100 (27.6) | 33 (33.0) | 67 (67.0) | **<0.001** |
| 12 - <24 months | 118 (32.5) | 83 (70.3) | 35 (29.7) |  |
| 24 - <60 months | 117 (32.2) | 91 (77.8) | 26 (22.2) |  |
| ≥60 months | 28 (7.7) | 9 (32.1) | 19 (67.9) |  |
| Median (IQR) years | 1.62 (2.1) | 1.93 (1.8) | 1.08 (2.2) | **<0.001** |
| Gender |  |  |  |  |
| Female | 159 (43.8) | 98 (61.6) | 61 (38.4) | 0.465 |
| Male | 204 (56.2) | 118 (57.8) | 86 (42.2) |  |
| Father’s indigenous status |  |  |  |  |
| Non-indigenous | 292 (80.4) | 183 (62.7) | 109 (37.3) | **0.044** |
| Indigenous | 64 (17.6) | 30 (46.9) | 34 (53.1) |  |
| Declined/unknown/missing | 7 (1.9) | 3 (42.9) | 4 (57.1) |  |
| Mother’s indigenous status |  |  |  |  |
| Non-indigenous | 283 (78.0) | 177 (62.5) | 106 (37.5) | **0.026** |
| Indigenous | 80 (22.0) | 39 (48.8) | 41 (51.3) |  |
| Child’s indigenous status |  |  |  |  |
| Non-indigenous | 261 (71.9) | 162 (62.1) | 99 (37.9) | 0.111 |
| Indigenous | 102 (28.1) | 54 (52.9) | 48 (47.1) |  |
| *If child is Indigenous,* |  |  |  |  |
| have cultural connection with home | 76 (74.5) | 39 (51.3) | 37 (48.7) | 0.636** |
| have cultural connection with traditional land | 41 (40.2) | 21 (51.2) | 20 (48.8) | 0.946 |
| have family members from Stolen Generation | 43 (42.2) | 25 (58.1) | 18 (41.9) | 0.337 |
| identified with an Aboriginal community | 68 (66.7) | 32 (47.1) | 36 (52.9) | 0.137** |
| Primary carer |  |  |  |  |
| Mother | 337 (92.8) | 199 (59.1) | 138 (41.0) | 0.529** |
| Father | 15 (4.1) | 11 (73.3) | 4 (26.7) |  |
| Others | 9 (2.5) | 5 (55.6) | 4 (44.4) |  |
| Missing | 2 (0.6) | 1 (50.0) | 1 (50.0) |  |
| Care type at home |  |  |  |  |
| Both parents at home | 273 (75.2) | 164 (60.1) | 109 (39.9) | 0.672** |
| Single parent at home | 67 (18.5) | 37 (55.2) | 30 (44.8) |  |
| Others | 20 (5.5) | 13 (65.0) | 7 (35.0) |  |
| Missing | 3 (0.8) | 2 (66.7) | 1 (33.3) |  |
| Duration at first symptoms (days) |  |  |  |  |
| <7 | 200 (55.1) | 124 (62.0) | 76 (38.0) | 0.197 |
| 7 - <14 | 61 (16.8) | 30 (49.2) | 31 (50.8) |  |
| 14 and above | 102 (28.1) | 62 (60.8) | 40 (39.2) |  |
| Median (IQR) | 5 (11) | 5 (11) | 6 (10) | 0.342 |
| Cough duration (days) |  |  |  |  |
| <14 | 266 (73.3) | 162 (60.9) | 104 (39.1) | **0.005**** |
| 14 - <28 | 41 (11.3) | 16 (39.0) | 25 (60.9) |  |
| ≥28 | 53 (14.6) | 38 (71.7) | 15 (28.3) |  |
| Declined/unknown/missing | 3 (0.8) | 0 (0.0) | 3 (100.0) |  |
| Cough troublesome score |  |  |  |  |
| Median (IQR) | 6 (4) | 6 (4) | 6 (4) | 0.908 |
| Cough lasting > 4 weeks |  |  |  |  |
| No | 236 (65.0) | 124 (52.5) | 112 (47.5) | **0.001** |
| Yes | 120 (33.1) | 88 (73.3) | 32 (26.7) |  |
| Declined/unknown/missing | 7 (1.9) | 4 (57.1) | 3 (42.9) |  |
| Diagnosed with any respiratory illness in the past 12 months | | |  |  |
| No | 167 (46.0) | 82 (49.1) | 85 (50.9) | **<0.001**** |
| Yes | 194 (53.4) | 133 (68.6) | 61 (31.4) |  |
| Unknown | 2 (0.6) | 1 (50.0) | 14 (50.0) |  |
| *If yes, what type & frequency?* |  |  |  |  |
| Asthma |  |  |  |  |
| No | 154 (79.4) | 104 (67.5) | 50 (32.5) | 0.547 |
| Yes | 40 (20.6) | 29 (72.5) | 11 (27.5) |  |
| Median (IQR) | 1 (1) | 1 (1) | 1 (0) | 0.495 |
| Pneumonia |  |  |  |  |
| No | 178 (91.8) | 122 (68.5) | 56 (31.5) | 0.986 |
| Yes | 16 (8.3) | 11 (68.8) | 5 (31.3) |  |
| Median (IQR) | 1 (0) | 1 (0) | 1 (0) | 1.000 |
| Flu |  |  |  |  |
| No | 183 (94.3) | 124 (67.8) | 59 (32.2) | 0.507* |
| Yes | 11 (5.7) | 9 (81.8) | 2 (18.2) |  |
| Median (IQR) | 1 (0) | 1 (0) | 1 (0) | 0.814 |
| Bronchiolitis |  |  |  |  |
| No | 131 (67.5) | 95 (72.5) | 36 (27.5) | 0.087 |
| Yes | 63 (32.5) | 38 (60.3) | 25 (39.7) |  |
| Median (IQR) | 1 (1) | 1 (1) | 1 (0) | 0.250 |
| Bronchitis |  |  |  |  |
| No | 168 (86.6) | 113 (67.3) | 55 (32.7) | 0.323 |
| Yes | 26 (13.4) | 20 (76.9) | 6 (23.1) |  |
| Median (IQR) | 1 (0) | 1 (1) | 1 (0) | 0.703 |
| Croup |  |  |  |  |
| No | 138 (71.1) | 86 (62.3) | 52 (37.7) | **0.003** |
| Yes | 56 (28.9) | 47 (83.9) | 9 (16.1) |  |
| Median (IQR) | 1 (1.5) | 1 (1.5) | 1.5 (1.5) | 0.723 |
| Whooping cough |  |  |  |  |
| No | 193 (99.5) | 132 (68.4) | 61 (31.6) | 1.000* |
| Yes | 1 (0.5) | 1 (100.0) | 0 (0.0) |  |
| Median (IQR) | 1 (0) | 1 (0) | - | - |
| Otitis media |  |  |  |  |
| No | 139 (71.6) | 87 (62.6) | 52 (37.4) | **0.004** |
| Yes | 55 (28.4) | 46 (83.6) | 9 (16.4) |  |
| Median (IQR) | 1 (1) | 1 (1) | 1 (1) | 0.862 |
| Tonsillitis |  |  |  |  |
| No | 157 (80.9) | 103 (65.6) | 54 (34.4) | 0.068 |
| Yes | 37 (19.1) | 30 (81.1) | 7 (18.9) |  |
| Median (IQR) | 1 (1) | 1 (1) | 1 (0) | 0.201 |
| Other |  |  |  |  |
| No | 152 (78.4) | 101 (66.4) | 51 (33.6) | 0.229 |
| Yes | 42 (21.6) | 32 (76.2) | 10 (23.8) |  |
| Median (IQR) | 1 (1) | 1 (1) | 1 (0) | 0.124 |
| Hospitalised in the past 12 months |  |  |  |  |
| No | 292 (80.4) | 168 (57.5) | 124 (42.5) | 0.121 |
| Yes | 71 (19.6) | 48 (67.6) | 23 (32.4) |  |
| Allergies history |  |  |  |  |
| No | 295 (81.3) | 169 (57.3) | 126 (42.7) | 0.200 |
| Yes | 61 (16.8) | 42 (68.8) | 19 (31.2) |  |
| Unknown | 7 (1.9) | 5 (71.4) | 2 (28.6) |  |
| Itchy rash duration > 6 months |  |  |  |  |
| No | 319 (87.9) | 192 (60.2) | 127 (39.8) | 0.501** |
| Yes | 42 (11.6) | 23 (54.8) | 19 (45.2) |  |
| Unknown/missing | 2 (0.5) | 1 (50.0) | 1 (50.0) |  |
| Eczema history |  |  |  |  |
| No | 297 (81.8) | 176 (59.3) | 121 (40.7) | **0.013** |
| Yes | 58 (16.0) | 39 (67.2) | 19 (32.8) |  |
| Declined/unknown/missing | 8 (2.2) | 1 (12.5) | 7 (87.5) |  |
| Wheezing/whistling history (in last 12 months) | |  |  |  |
| No | 156 (43.0) | 92 (59.0) | 64 (41.0) | 0.757** |
| Yes | 203 (55.9) | 123 (60.6) | 80 (39.4) |  |
| Declined/unknown/missing | 4 (1.1) | 1 (25.0) | 3 (75.0) |  |
| Other medical conditions |  |  |  |  |
| No | 298 (82.1) | 169 (56.7) | 129 (43.3) | **0.025**** |
| Yes | 64 (17.6) | 46 (71.9) | 18 (28.1) |  |
| Unknown /missing | 1 (0.3) | 1 (100.0) | 0 (0.0) |  |
| Number of children living in the house | |  |  |  |
| 0 | 114 (31.4) | 83 (72.8) | 31 (27.2) | **<0.001** |
| 1-2 | 208 (57.3) | 116 (55.8) | 92 (44.2) |  |
| 3-4 | 33 (9.1) | 11 (33.3) | 22 (66.7) |  |
| 5 and above | 8 (2.2) | 6 (75.0) | 2 (25.0) |  |
| Number of people staying in the same room with child | | |  |  |
| 0 | 147 (40.5) | 97 (66.0) | 50 (34.0) | **0.056**** |
| 1-2 | 185 (51.0) | 107 (57.8) | 78 (42.2) |  |
| 3-4 | 27 (7.4) | 11 (40.7) | 16 (59.3) |  |
| 5 and above | 3 (0.8) | 1 (33.3) | 2 (66.7) |  |
| Missing | 1 (0.3) | 0 (0.0) | 1 (100.0) |  |
| Number of people in house |  |  |  |  |
| 1-2 | 111 (30.6) | 78 (70.3) | 33 (29.7) | **0.012**** |
| 3-4 | 188 (51.8) | 108 (57.4) | 80 (42.6) |  |
| 5 and above | 62 (17.1) | 30 (48.4) | 32 (51.6) |  |
| Declined/unknown/missing | 2 (0.6) | 0 (0.0) | 2 (100.0) |  |
| House condition |  |  |  |  |
| Excellent | 151 (41.6) | 96 (63.6) | 55 (36.4) | **0.038**** |
| Good | 142 (39.1) | 87 (61.3) | 55 (38.7) |  |
| Average | 54 (14.9) | 30 (55.6) | 24 (44.4) |  |
| Poor | 13 (3.6) | 3 (23.1) | 10 (76.9) |  |
| Very poor | 1 (0.3) | 0 (0.0) | 1 (100.0) |  |
| Missing | 2 (0.6) | 0 (0.0) | 2 (100.0) |  |
| Mould in house |  |  |  |  |
| No | 291 (80.2) | 181 (62.2) | 110 (37.8) | **0.047**** |
| Yes | 71 (19.6) | 35 (49.3) | 36 (50.7) |  |
| Missing | 1 (0.3) | 0 (0.0) | 1 (100.0) |  |
| Mother's age at birth (years) |  |  |  |  |
| Mean (sd) | 29.0 (6.4) | 29.1 (6.2) | 28.7 (6.6) | 0.528 |
| Father's age at birth (years) |  |  |  |  |
| Mean (sd) | 31.9 (7.1) | 31.8 (7.2) | 32.0 (6.9) | 0.760 |
| Mother's age at enrolment (years) |  |  |  |  |
| Mean (sd) | 31.1 (6.6) | 31.4 (6.3) | 30.6 (7.0) | 0.241 |
| Father's age at enrolment (years) |  |  |  |  |
| Mean (sd) | 33.9 (7.1) | 34.0 (7.2) | 33.9 (7.1) | 0.861 |
| Birth weight (grams) |  |  |  |  |
| Underweight (<2500) | 24 (6.6) | 16 (66.7) | 8 (33.3) | 0.459 |
| Healthy weight (=>2500) | 339 (93.4) | 200 (59.0) | 139 (41.0) |  |
| Gestational age (weeks) |  |  |  |  |
| Full-term (≥37 weeks) | 329 (90.6) | 193 (58.7) | 136 (41.3) | 0.310 |
| Pre-term (<37 weeks) | 34 (9.4) | 23 (67.7) | 11 (32.3) |  |
| Received flu vaccine during pregnancy | | | |  |
| No | 172 (47.4) | 100 (58.1) | 72 (41.9) | 0.868 |
| Yes | 154 (42.4) | 94 (61.0) | 60 (39.0) |  |
| Declined/unknown/missing | 37 (10.2) | 22 (59.5) | 15 (40.5) |  |
| Received pertussis vaccine during pregnancy | | | |  |
| No | 92 (25.3) | 49 (53.2) | 43 (46.7) | 0.346 |
| Yes | 232 (63.9) | 144 (62.1) | 88 (37.9) |  |
| Declined/unknown/missing | 39 (10.7) | 23 (59.0) | 16 (41.0) |  |
| Child admitted to neonatal ICU post birth for problems with breathing | | | |  |
| No | 283 (78.0) | 164 (58.0) | 119 (42.0) | 0.304** |
| Yes | 76 (20.9) | 49 (64.5) | 27 (35.5) |  |
| Declined/unknown/missing | 4 (1.1) | 3 (75.0) | 1 (25.0) |  |
| Maternal smoking during pregnancy |  |  |  |  |
| No | 298 (82.1) | 179 (60.1) | 119 (39.9) | 0.508** |
| Yes | 63 (17.4) | 35 (55.6) | 28 (44.4) |  |
| Declined/unknown/missing | 2 (0.6) | 2 (100.0) | 0 (0.0) |  |
| Any smoke exposure |  |  |  |  |
| No | 251 (69.2) | 146 (58.2) | 105 (41.8) | 0.475** |
| Yes | 111 (30.6) | 69 (62.2) | 42 (37.8) |  |
| Declined/unknown/missing | 1 (0.3) | 1 (100.0) | 0 (0.0) |  |
| Family history of asthma |  |  |  |  |
| No | 112 (30.8) | 67 (59.8) | 148 (59.2) | 0.911** |
| Yes | 250 (68.9) | 148 (59.2) | 102 (40.8) |  |
| Declined/unknown/missing | 1 (0.3) | 1 (100.0) | 0 (0.0) |  |
| Family history of lung disease |  |  |  |  |
| No | 274 (75.5) | 167 (61.0) | 107 (39.0) | 0.617 |
| Yes | 80 (22.0) | 44 (55.0) | 36 (45.0) |  |
| Declined/unknown/missing | 9 (2.5) | 5 (55.6) | 4 (44.4) |  |
| Family history of allergies |  |  |  |  |
| No | 184 (50.7) | 100 (54.4) | 84 (45.6) | 0.127 |
| Yes | 171 (47.1) | 111 (64.9) | 60 (35.1) |  |
| Declined/unknown/missing | 8 (2.2) | 5 (62.5) | 3 (37.5) |  |
| Household pets |  |  |  |  |
| No | 168 (46.3) | 93 (55.4) | 75 (44.6) | 0.129** |
| Yes | 193 (53.1) | 122 (63.2) | 71 (36.8) |  |
| Declined/unknown/missing | 2 (0.6) | 1 (50.0) | 1 (50.0) |  |
| Received flu vaccine in past 12 mouths | | | |  |
| No | 243 (66.9) | 157 (64.6) | 86 (35.4) | **<0.001** |
| Yes | 58 (16.0) | 37 (63.8) | 21 (36.2) |  |
| Unknown/missing | 26 (7.2) | 16 (61.5) | 10 (38.5) |  |
| Not applicable | 36 (9.9) | 6 (16.7) | 30 (83.3) |  |
| Age appropriately immunised |  |  |  |  |
| Yes | 336 (92.6) | 204 (60.7) | 132 (39.3) | 0.186 |
| No | 20 (5.5) | 8 (40.0) | 12 (60.0) |  |
| Declined/unknown/missing | 7 (1.9) | 4 (57.1) | 3 (42.9) |  |
| Cough knowledge: cough longer than 4 weeks | | | |  |
| Abnormal | 345 (95.0) | 206 (59.7) | 139 (40.3) | 0.815 |
| Normal | 10 (2.8) | 5 (50.0) | 5 (50.0) |  |
| Declined/unknown/missing | 8 (2.2) | 5 (62.5) | 3 (37.5) |  |
| Cough knowledge: wet sounding cough | | | |  |
| Abnormal | 295 (81.3) | 171 (58.0) | 124 (42.0) | 0.450 |
| Normal | 46 (12.7) | 30 (65.2) | 16 (34.8) |  |
| Declined/unknown/missing | 22 (6.1) | 15 (68.2) | 7 (31.8) |  |
| Cough knowledge: use antibiotics for cold/flu | | | |  |
| No | 187 (51.5) | 121 (64.7) | 66 (35.3) | **0.024** |
| Maybe | 99 (27.3) | 60 (60.6) | 39 (39.4) |  |
| Yes | 61 (16.8) | 26 (42.6) | 35 (57.4) |  |
| Declined/unknown/missing | 16 (4.4) | 9 (56.3) | 7 (43.7) |  |
| Cough knowledge: duration of a course of antibiotics | | | |  |
| Until they are all finished | 316 (87.1) | 186 (58.9) | 130 (41.1) | 0.429 |
| Until no more symptoms | 34 (9.4) | 20 (58.8) | 14 (41.2) |  |
| Declined/unknown/missing | 13 (3.6) | 10 (76.9) | 3 (23.1) |  |
| Medications given in past 7 days |  |  |  |  |
| No | 57 (15.7) | 25 (43.9) | 32 (56.1) | **0.009** |
| Yes | 306 (84.3) | 191 (62.4) | 115 (37.6) |  |
| Receiving government welfare benefits | |  |  |  |
| Yes | 141 (38.8) | 78 (55.3) | 63 (44.7) | **0.031** |
| No | 216 (59.5) | 137 (63.4) | 79 (36.6) |  |
| Declined/unknown/missing | 6 (1.7) | 1 (16.7) | 5 (83.3) |  |
| Private insurance covering children |  |  |  |  |
| No | 228 (62.8) | 123 (54.0) | 105 (46.0) | **0.016** |
| Yes | 128 (35.3) | 89 (69.5) | 39 (30.5) |  |
| Declined/unknown/missing | 7 (1.9) | 4 (57.1) | 3 (42.9) |  |
| Employment of Father |  |  |  |  |
| Unemployed | 43 (11.8) | 16 (37.2) | 27 (62.8) | **0.005** |
| Employed PT/casual | 31 (8.5) | 17 (54.8) | 14 (45.2) |  |
| Employed FT | 247 (68.0) | 161 (65.2) | 86 (34.8) |  |
| Not applicable | 17 (4.7) | 11 (64.7) | 6 (35.3) |  |
| Declined/unknown/missing | 25 (6.9) | 11 (44.0) | 14 (56.0) |  |
| Employment of Mother |  |  |  |  |
| Unemployed | 157 (43.2) | 64 (40.8) | 93 (59.2) | **<0.001** |
| Employed PT/casual | 109 (30.0) | 83 (76.1) | 26 (23.9) |  |
| Employed FT | 82 (22.6) | 63 (76.8) | 19 (23.2) |  |
| Not applicable | 5 (1.4) | 3 (60.0) | 2 (40.0) |  |
| Declined/unknown/missing | 10 (2.8) | 3 (30.0) | 7 (70.0) |  |
| Household Income |  |  |  |  |
| <$52000 | 96 (26.4) | 46 (47.9) | 50 (52.1) | **0.001** |
| $52000 - <$78000 | 33 (9.1) | 19 (57.6) | 14 (42.4) |  |
| $78000 - < $104000 | 50 (13.8) | 38 (76.0) | 12 (24.0) |  |
| $104000 - <$156000 | 61 (16.8) | 42 (68.9) | 19 (31.1) |  |
| $156000 - <$200000 | 28 (7.7) | 19 (67.9) | 9 (32.1) |  |
| >= $200000 | 20 (5.5) | 16 (80.0) | 4 (20.0) |  |
| Declined/unknown/missing | 75 (20.7) | 36 (48.0) | 39 (52.0) |  |
| Highest education of mother |  |  |  |  |
| High school (or lower) | 138 (38.0) | 78 (56.5) | 60 (43.5) | 0.178 |
| Post school | 74 (20.4) | 43 (58.1) | 31 (41.9) |  |
| Tertiary | 136 (37.5) | 89 (65.4) | 47 (34.6) |  |
| Declined/unknown/missing | 15 (4.1) | 6 (40.0) | 9 (60.0) |  |
| Highest education of father |  |  |  |  |
| High school (or lower) | 133 (36.6) | 80 (60.1) | 53 (39.9) | **0.020** |
| Post school | 85 (23.4) | 49 (57.7) | 36 (42.3) |  |
| Tertiary | 94 (25.9) | 62 (66.0) | 32 (34.0) |  |
| N/A | 12 (3.3) | 10 (83.3) | 2 (16.7) |  |
| Declined/unknown/missing | 39 (10.7) | 15 (38.5) | 24 (61.5) |  |

*Fisher exact test used instead of Chi-square

**Response of “N/A”, “declined”, “unknown” & “missing” were excluded from the test for significance

***Supplementary Table 2 – Adjusted odd ratios for characteristics associated with childcare attendance on a regular basis, n=363***

|  | aOR | 95% CI | *P*-value |
| --- | --- | --- | --- |
| **Enrolment site** |  |  |  |
| Brisbane | Ref |  |  |
| Caboolture | 1.88 | 0.97 – 3.62 | 0.060 |
| Toowoomba / Warwick | 0.33 | 0.13 – 0.87 | **0.025** |
| **Age of enrolment (months)** |  |  |  |
| ≥60 | Ref |  |  |
| 24 - <60 | 13.66 | 4.51 – 41.34 | **<0.001** |
| 12 - <24 | 8.08 | 2.76 – 23.67 | **<0.001** |
| <12 | 1.49 | 0.51 – 4.33 | 0.462 |
| **History of chronic cough** |  |  |  |
| No | Ref |  |  |
| Yes | 2.22 | 1.19 – 4.16 | **0.013** |
| Declined/unknown/missing | 0.79 | 0.09 – 6.61 | 0.826 |
| **History of croup** |  |  |  |
| No | Ref |  |  |
| Yes | 3.56 | 1.45 – 8.74 | **0.006** |
| **Number of children living in the house** |  |  |  |
| 0 | Ref |  |  |
| 1-2 | 0.45 | 0.24 – 0.84 | **0.012** |
| 3-4 | 0.49 | 0.17 – 1.42 | 0.190 |
| 5 and above | 3.44 | 0.41 – 29.08 | 0.257 |
| **Employment of mother** |  |  |  |
| Unemployed | Ref |  |  |
| Employed PT/casual | 6.48 | 3.18 – 13.21 | **<0.001** |
| Employed FT | 6.59 | 3.03 – 14.35 | **<0.001** |
| Not applicable | 5.17 | 0.45 – 59.72 | 0.188 |
| Declined/unknown/missing | 0.54 | 0.12 – 2.44 | 0.424 |

***Supplementary Table 3 – Selected* baseline characteristics comparing children with and without CC, and with unknown cough duration, n =362, n (%)***

| Characteristic | All children (n=362) | Chronic cough  Yes  (n=95) | Chronic cough  No  (n=182) | Chronic cough  Unknown (n=85) | p-value |
| --- | --- | --- | --- | --- | --- |
| Enrolment site |  |  |  |  |  |
| Brisbane | 205 (56.6) | 46 (22.4) | 108 (52.7) | 51 (24.9) | 0.527 |
| Caboolture | 115 (31.8) | 36 (31.3) | 53 (46.1) | 26 (22.6) |  |
| Toowoomba / Warwick | 42 (11.6) | 13 (30.9) | 21 (50.0) | 8 (19.1) |  |
| Age at enrolment |  |  |  |  |  |
| <12 months | 100 (27.6) | 29 (29.0) | 46 (46.0) | 25 (25.0) | 0.323 |
| 12 - <24 months | 117 (32.3) | 34 (29.1) | 62 (53.0) | 21 (18.0) |  |
| 24 - <60 months | 117 (32.3) | 29 (24.8) | 56 (47.9) | 32 (27.4) |  |
| ≥60 months | 28 (7.7) | 3 (10.7) | 18 (64.3) | 7 (25.0) |  |
| Median (IQR) years | 1.62 (2.1) | 1.68 (2.1) | 1.51 (1.7) | 1.93 (2.1) | 0.390 |
| Gender |  |  |  |  |  |
| Female | 158 (43.6) | 46 (29.1) | 76 (48.1) | 36 (22.8) | 0.467 |
| Male | 204 (56.4) | 49 (24.0) | 106 (52.0) | 49 (24.0) |  |
| Child’s indigenous status |  |  |  |  |  |
| Non-indigenous | 260 (71.8) | 65 (25.0) | 133 (51.2) | 62 (23.8) | 0.594 |
| Indigenous | 102 (28.2) | 30 (29.4) | 49 (48.0) | 23 (22.6) |  |
| Season on enrolment |  |  |  |  |  |
| Spring | 89 (24.6) | 25 (28.1) | 45 (50.6) | 19 (21.3) | **0.008** |
| Summer | 43 (11.9) | 6 (14.0) | 26 (60.5) | 11 (25.6) |  |
| Autumn | 89 (24.6) | 19 (21.4) | 51 (57.3) | 19 (21.3) |  |
| Winter | 141 (39.0) | 45 (31.9) | 60 (42.6) | 36 (25.5) |  |
|  |  |  |  |  |  |
| Duration at first symptoms (days) | |  |  |  |  |
| <7 | 199 (55.0) | 36 (18.1) | 115 (57.8) | 48 (24.1) | **0.007** |
| 7 - <14 | 61 (16.9) | 20 (32.8) | 25 (41.0) | 16 (26.2) |  |
| 14 and above | 102 (28.2) | 39 (38.2) | 42 (41.2) | 21 (20.6) |  |
| Median (IQR) | 5 (11) | 8 (18) | 5 (7) | 5 (10) | **0.001** |
| Cough duration (days) |  |  |  |  |  |
| <14 | 265 (73.2) | 60 (22.6) | 144 (54.3) | 61 (23.0) | **0.007** |
| 14 - <28 | 41 (11.3) | 9 (22.0) | 20 (48.8) | 12 (29.3) |  |
| ≥28 | 53 (14.6) | 26 (49.1) | 16 (30.2) | 11 (20.8) |  |
| Declined/unknown/missing** | 3 (0.8) | 0 (0.0) | 2 (66.7) | 1 (33.3) |  |
| Cough troublesome score |  |  |  |  |  |
| Median (IQR) | 6 (4) | 6 (3) | 6 (4) | 7 (4) | **0.002** |
| History of chronic cough |  |  |  |  |  |
| No | 235 (64.9) | 50 (21.3) | 135 (57.5) | 50 (21.2) | **0.000** |
| Yes | 120 (33.2) | 44 (36.7) | 42 (35.0) | 34 (28.3) |  |
| Declined/unknown/missing | 7 (1.9) | 1 (14.3) | 5 (71.4) | 1 (14.3) |  |
| Diagnosed with any respiratory illness in the past 12 months | | | | |  |
| No | 166 (45.9) | 42 (25.3) | 94 (56.6) | 30 (18.1) | **0.026** |
| Yes | 194 (53.6) | 53 (27.3) | 87 (44.9) | 54 (27.8) |  |
| Unknown** | 2 (0.6) | 0 (0.0) | 1 (50.0) | 1 (50.0) |  |
| Itchy rash duration > 6 months |  |  |  |  |  |
| No | 318 (87.9) | 79 (24.8) | 166 (52.2) | 73 (23.0) | **0.024** |
| Yes | 42 (11.6) | 16 (38.1) | 14 (33.3) | 12 (28.6) |  |
| Unknown/missing | 2 (0.6) | 0 (0.0) | 2 (100.0) | 0 (0.0) |  |
| Eczema history |  |  |  |  |  |
| No | 296 (81.8) | 73 (24.7) | 157 (53.0) | 66 (22.3) | 0.087 |
| Yes | 58 (16.0) | 21 (36.2) | 22 (37.9) | 15 (25.9) |  |
| Declined/unknown/missing | 8 (2.2) | 1 (12.5) | 3 (37.5) | 4 (50.0) |  |
| History of wheezing/whistling |  |  |  |  |  |
| No | 155 (42.8) | 26 (16.8) | 93 (60.0) | 36 (23.2) | **0.001** |
| Yes | 203 (56.1) | 69 (34.0) | 86 (42.4) | 48 (23.6) |  |
| Declined/unknown/missing** | 4 (1.1) | 0 (0.0) | 3 (75.0) | 1 (25.0) |  |
| History of other medical conditions | | | | |  |
| No | 297 (82.0) | 67 (22.6) | 158 (53.2) | 72 (24.2) | **0.024** |
| Yes | 64 (17.7) | 27 (42.2) | 24 (37.5) | 13 (20.3) |  |
| Unknown/missing** | 1 (0.3) | 1 (100.0) | 0 (0.0) | 0 (0.0) |  |
| Number of other children living in the house | | | | |  |
| 0 | 113 (31.2) | 35 (31.0) | 44 (38.9) | 34 (30.1) | **0.018** |
| 1-2 | 208 (57.5) | 48 (23.1) | 116 (55.8) | 44 (21.2) |  |
| 3-4 | 33 (9.1) | 12 (36.4) | 16 (48.5) | 5 (15.2) |  |
| 5 and above | 8 (2.2) | 0 (0.0) | 6 (75.0) | 2 (25.0) |  |
| Number of people in house |  |  |  |  |  |
| 1-2 | 110 (30.4) | 37 (33.6) | 43 (39.1) | 30 (27.3) | **0.017** |
| 3-4 | 188 (51.9) | 44 (23.4) | 100 (53.2) | 44 (23.4) |  |
| 5 and above | 62 (17.1) | 14 (22.6) | 37 (59.7) | 11 (17.7) |  |
| Declined/unknown/missing** | 2 (0.6) | 0 (0.0) | 2 (100.0) | 0 (0.0) |  |
| House condition |  |  |  |  |  |
| Excellent | 151 (41.7) | 35 (23.2) | 85 (56.3) | 31 (20.5) | **0.061** |
| Good | 141 (39.0) | 38 (27.0) | 60 (42.5) | 43 (30.5) |  |
| Average | 54 (14.9) | 20 (37.0) | 26 (48.2) | 8 (14.8) |  |
| Poor | 13 (3.6) | 1 (7.7) | 9 (69.2) | 3 (23.1) |  |
| Very poor | 1 (0.3) | 1 (100.0) | 0 (0.0) | 0 (0.0) |  |
| Missing** | 2 (0.6) | 0 (0.0) | 2 (100.0) | 0 (0.0) |  |
| Mother's age at birth (years) |  |  |  |  |  |
| Mean (sd) | 28.94 (6.4) | 29.43 (7.0) | 29.55 (5.9) | 27.08 (6.4) | **0.009** |
| Mother’s age at enrolment (years) | | | | |  |
| Mean (sd) | 31.04 (6.6) | 31.32 (6.9) | 31.73 (6.3) | 29.28 (6.6) | **0.016** |
| Gestational age (weeks) |  |  |  |  |  |
| Full-term (≥37 weeks) | 328 (90.6) | 79 (24.1) | 171 (52.1) | 78 (23.8) | **0.032** |
| Pre-term (<37 weeks) | 34 (9.4) | 16 (47.1) | 11 (32.4) | 7 (20.6) |  |
| Received flu vaccine in pregnancy |  |  |  |  |  |
| No | 172 (47.5) | 39 (22.7) | 93 (54.1) | 40 (23.3) | **0.092** |
| Yes | 153 (42.3) | 49 (32.0) | 67 (43.8) | 37 (24.2) |  |
| Declined/unknown/missing | 37 (10.2) | 7 (18.9) | 22 (59.5) | 8 (21.6) |  |
| Received pertussis vaccine during pregnancy | |  |  |  |  |
| No | 92 (25.4) | 17 (18.5) | 55 (59.8) | 20 (21.7) | **0.072** |
| Yes | 231 (63.8) | 71 (30.7) | 106 (45.9) | 54 (23.4) |  |
| Declined/unknown/missing | 39 (10.8) | 7 (17.9) | 21 (53.9) | 11 (28.2) |  |
| Attend childcare |  |  |  |  |  |
| No | 147 (40.6) | 31 (21.1) | 85 (57.8) | 31 (21.1) | **0.018** |
| Yes | 215 (59.4) | 64 (29.8) | 97 (45.1) | 54 (25.1) |  |
| Private insurance covering children | | | | |  |
| No | 228 (63.0) | 60 (26.3) | 104 (45.6) | 64 (28.1) | **0.070** |
| Yes | 127 (35.1) | 34 (26.8) | 74 (58.3) | 19 (15.0) |  |
| Declined/unknown/missing | 7 (1.9) | 1 (14.3) | 4 (57.1) | 2 (28.6) |  |

** Only variables with a p-value of <0.1 presented.*

*** Category has been excluded from p-value calculation*

***Supplementary Table 4 – Adjusted risk ratios for children who developed chronic cough at Day 28 following enrolment, n=350 (adjusted for age and gender)***

|  | *Cough persistence at day 28* | | | *Unknown cough persistence at day 28* | | |
| --- | --- | --- | --- | --- | --- | --- |
|  | aRR | 95% CI | *P*-value | aRR | 95% CI | *P*-value |
| **Duration at first symptoms (days)** |  |  |  |  |  |  |
| <7 | Ref |  |  | Ref |  |  |
| 7 - <14 | 2.79 | 1.29 – 6.06 | **0.009** | 1.86 | 0.84 – 4.12 | 0.128 |
| 14 and above | 2.60 | 1.37 – 4.92 | **0.003** | 1.28 | 0.63 – 2.58 | 0.493 |
|  |  |  |  |  |  |  |
| **Cough troublesome score** | 1.06 | 0.93 – 1.20 | 0.390 | 1.26 | 1.11 – 1.44 | **<0.001** |
|  |  |  |  |  |  |  |
| **History of respiratory illness diagnosis** |  |  |  |  |  |  |
| No | Ref |  |  | Ref |  |  |
| Yes | 0.74 | 0.40 – 1.39 | 0.351 | 2.11 | 1.11 – 4.03 | **0.023** |
| **History of wheezing/whistling** |  |  |  |  |  |  |
| No | Ref |  |  | Ref |  |  |
| Yes | 2.57 | 1.38 – 4.79 | **0.003** | 0.89 | 0.48 – 1.66 | 0.718 |
| **History of other medical conditions** |  |  |  |  |  |  |
| No | Ref |  |  | Ref |  |  |
| Yes | 2.71 | 1.29 – 5.69 | **0.009** | 0.81 | 0.35 – 1.85 | 0.615 |
| **Number of other children living in the house** |  |  |  |  |  |  |
| 0 | Ref |  |  | Ref |  |  |
| 1-2 | 0.54 | 0.29 – 1.02 | 0.057 | 0.50 | 0.26 – 0.96 | **0.036** |
| 3-4 | 1.08 | 0.40 – 2.96 | 0.876 | 0.46 | 0.14 – 1.53 | 0.206 |
| 5 and above | n/a | n/a | 0.980 | 0.57 | 0.10 – 3.41 | 0.538 |
|  |  |  |  |  |  |  |
| **Mother’s age at enrolment (years)** | 1.00 | 0.96 – 1.06 | 0.680 | 0.94 | 0.90 – 1.00 | **0.032** |
|  |  |  |  |  |  |  |
| **Gestational age (weeks)** |  |  |  |  |  |  |
| ≥37 weeks | Ref |  |  | Ref |  |  |
| <37 weeks | 3.54 | 1.43 – 8.75 | **0.006** | 1.24 | 0.42 – 3.66 | 0.698 |
| **Attend childcare** |  |  |  |  |  |  |
| No | Ref |  |  | Ref |  |  |
| Yes | 2.04 | 1.10 – 3.76 | **0.023** | 1.64 | 0.87 – 3.08 | 0.123 |
| **Private insurance covering children** |  |  |  |  |  |  |
| No | Ref |  |  | Ref |  |  |
| Yes | 0.82 | 0.43 – 1.57 | 0.546 | 0.45 | 0.22 – 0.93 | **0.032** |
| Declined/unknown/missing | 0.42 | 0.04 – 4.55 | 0.473 | 0.29 | 0.03 – 3.12 | 0.310 |

***Supplementary Table 5 – Persistent cough by childcare attendance over 4 weeks of observation.***

|  | Cough persisted 1 week | | Cough persisted 2 weeks | | Cough persisted 3 weeks | | Cough persisted 4 weeks | |
| --- | --- | --- | --- | --- | --- | --- | --- | --- |
|  | Successful contact = 283/362 | | Successful contact = 253/362 | | Successful contact = 245/362 | | Successful contact = 247/362 | |
|  | Yes | No | Yes | No | Yes | No | Yes | No |
| Not attending Childcare | 100 (86.2) | 16 (13.8) | 49 (51.0) | 47 (49.0) | 40 (40.4) | 59 (59.6) | 30 (19.1) | 73 (70.9) |
| Attending Childcare | 152 (91.0) | 15 (9.0) | 94 (59.9) | 63 (40.1) | 66 (45.2) | 80 (54.8) | 60 (41.7) | 84 (58.3) |
| Total children per week | 252 (89.0) | 31 (11.0) | 143 (56.5) | 110 (43.5) | 106 (43.3) | 139 (56.7) | 90 (36.4) | 157 (63.6) |
| P-value | 0.202 | | 0.169 | | 0.457 | | **0.043** | |
